# Transplanting Teeth

**Published:** 1871-11

**Authors:** 


*Transplanting Teeth.*—In the "Am. Eclectic Medical Review" and "Medical Cosmos" we find the following:

Transplanting teeth, there is good authority for believing, has been performed successfully by several dental surgeons. Twenty years ago, a surgeon dntist in Ithaca, in this State, extracted the teeth of a person which had grown in sideways, and re-set them straight, the operation being quite successful. Recently, an English dentist of repute, a Dr. Coleman, has applied this process to the treatment of *periodontitis*, from which many persons suffer severely, and in frequent instances nearly or quite a sound tooth is extracted to afford relief. Mr. Coleman extracts the diseased tooth, cleans out the cavities, if there are any, and fills them ; scrapes the fangs clean from the diseased matter and accumulation of tartar which causes the pain, taking care, however, not to disturb the mucous membrane about the root of the tooth. He then bathes both the tooth and the cavity from which it was taken in a solution of carbolic acid, and returns the member to its place, using no mechanical appliance for making it keep its position, it becoming soon as firm as ever. These are singular statements, but are so well authenticated that they cannot be doubted.

Dr. L. D. Walter, (*Dental Times*,) reports a case of successful replanting of two superior incisor teeth accidentally dislodged by a fall, after they had become "perfectly dry and had been out of place for fifteen hours. The teeth were thoroughly cleaned, and after the alveoli had been syringed with tepid water, inserted, and held in position by means of the fingers for about twenty minutes. The crowns were then capped with sheet tin and this bound by means of fine wire to the adjoining laterals. The appliance was kept in position one month and then removed. In the mean time a suitable mouth-wash was used several times daily, to reduce the inflammation. The teeth are now, apparently, as firm and healthy as ever. They are not all decayed, and the color does not indicate death of the pulp. The above operation was performed eleven months ago."

